# Case Report: Congenital infiltrating lipomatosis of face

**DOI:** 10.4103/0971-3026.43847

**Published:** 2008-11

**Authors:** Rangasami Rajeswaran, Jyotsna Murthy, Anupama Chandrasekharan, Santhosh Joseph

**Affiliations:** Department of Radiology and Imaging Sciences, Sri Ramachandra Medical College and Research Institute, Sri Ramachandra University, Chennai - 600 116, India; 1 Department of Plastic Surgery, Sri Ramachandra Medical College and Research Institute, Sri Ramachandra University, Chennai - 600 116, India

**Keywords:** Congenital, lipomatosis

## Abstract

Congenital infiltrating lipomatosis of the face is a rare condition characterized by diffuse fatty infiltration of the facial soft tissues. There may be muscle involvement along with associated bony hyperplasia. It is a type of lipomatous tumor that is congenital in origin; it is rare and seen usually in childhood. We recently saw an 11-year-old girl with this condition. She presented with a swelling of the right side of the face that had been present since birth; there were typical findings on plain radiographs, CT, and MRI. The patient underwent cosmetic surgery. Histopathological examination showed mature adipocytes without any capsule.

Congenital infiltrating lipomatosis of the face is a rare entity characterized by collections of nonencapsulated, mature lipocytes that infiltrate local tissues, leading to craniofacial deformities.[1] These children have normal psychomotor development; the main concern is the esthetic appearance. Till date, fewer than 50 cases have been reported in the English literature.[2,3] We report the radiological findings in one such patient.

## Case Report

**Figure 1 (A, B):**
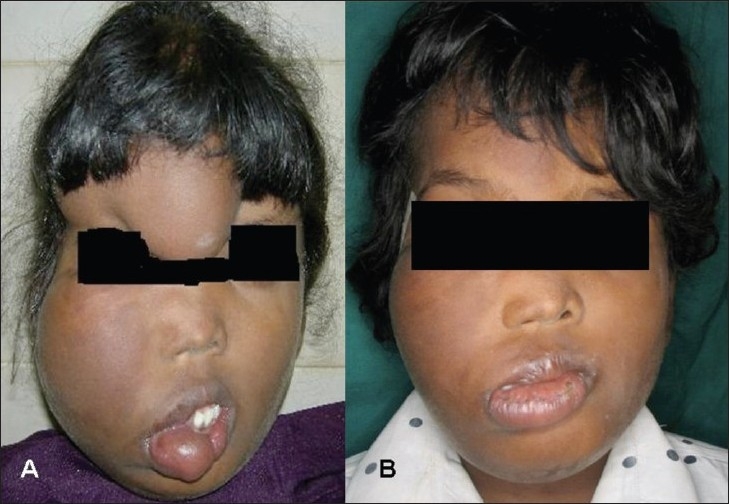
The appearance of the patient before (A) and after (B) surgery

An 11-year old girl presented with a swelling of the right side of the face since birth that had been progressively increasing in size [Figure 1]. There was no history of pain. On examination, a soft, nonpulsatile, noncompressible, ill-defined swelling was present over the face on the right side. The skin over the swelling was normal and there was no discoloration. There was no cervical lymphadenopathy.

**Figure 2:**
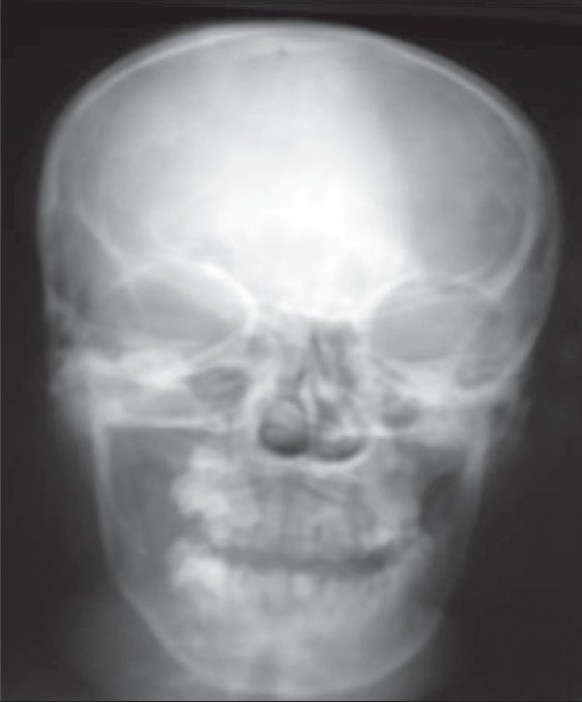
Plain frontal radiograph shows sclerosis of the right frontal and zygomatic bones

**Figure 3 (A–C):**
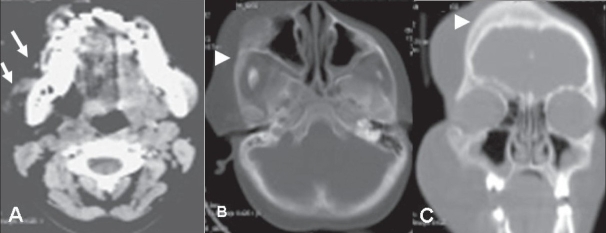
Axial soft tissue (A), axial bone window (B), and coronal bone window reconstruction (C) images show a fat-density lesion in the subcutaneous, muscular, and intermuscular planes of the face (arrows), with thickening of the right frontal and zygomatic bones (arrowheads)

Frontal skull radiographs [Figure 2] showed increased density of the right orbital and frontal bones. CT scan showed an ill-defined, inhomogeneous, fat-density infiltrative lesion involving the superficial as well as deep planes of the right hemiface and upper neck. The masticator, parapharyngeal, submandibular, and sublingual spaces on the right side were involved [Figure 3A]. The subcutaneous, muscular, and intermuscular planes were involved. The tongue and palate were also involved on the right side. The right orbital, maxillary, zygomatic, and frontal bones were thickened [Figures 3B and C].

**Figure 4 (A–D):**
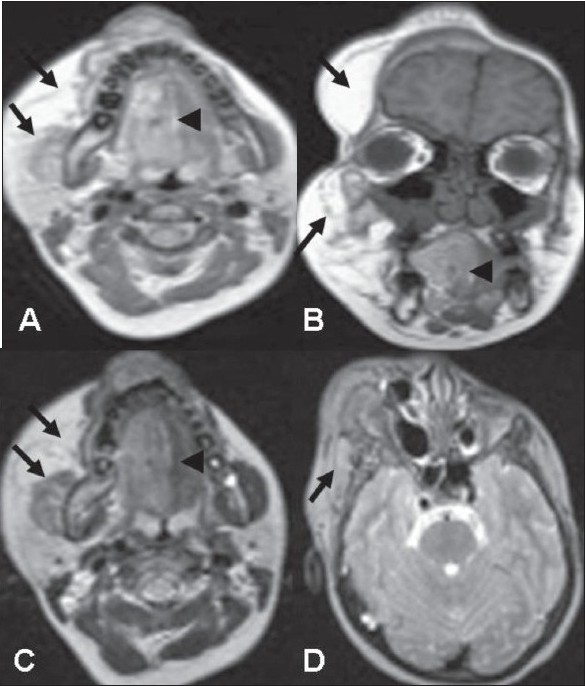
Axial T1W (A), coronal T1W (B), and axial T2W (C, D) images show an ill-defined lesion with fat-signal intensity (arrows) in the subcutaneous, muscular, and intermuscular planes of the face and neck. The tongue and palate on the right side are also involved (arrowheads)

**Figure 5 (A, B):**
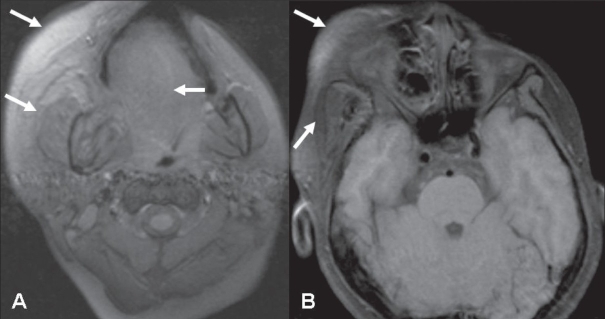
Fat-suppressed T1W axial images show hypointensity within the lesion, suggesting presence of fat (arrows)

Subsequent MRI on a 1.5-T scanner showed a nonenhancing, ill-defined, infiltrative, inhomogeneous, hyperintense lesion (isointense to fat) on T1W images in the subcutaneous, muscular, and intermuscular planes of the face and upper neck on the right side [Figures 4A and B]; the lesion appeared isointense to fat on T2W images [Figures 4C and D]. The lesion involved the masticator, parapharyngeal, submandibular, and sublingual spaces on the right side. The tongue and palate on the right side were also mildly hyperintense, suggesting involvement. The lesion was hypointense on fat-suppressed images [Figure 5]. A radiological diagnosis of congenital infiltrating lipomatosis of the face was made.

The patient underwent cosmetic surgery. Histopathological examination showed mature adipocytes without any capsule. Diffuse infiltration of the intervening muscles was also identified. There was no cell atypia to suggest malignancy. These findings confirmed the radiological diagnosis.

## Discussion

Congenital infiltrating lipomatosis was first described by Slavin *et al.*[1] in 1983. The etiology is unclear. This is a rare entity[2] and is classified as a subgroup of lipoma. Benign lipomas[4] have five subgroups:

1. Simple encapsulated lipoma
2. Lipoma variants like angiomyolipoma
3. Hamartomatous lesions
4. Infiltrating or diffuse lipomatosis
5. Benign tumor of brown fat – hibernoma

Infiltrating lipomas usually occur after the third decade of life[4] and involve subcutaneous tissue, muscles, and bones. When they have been present since birth or infancy, they are called congenital infiltrating lipomatosis and are commonly seen in the face.

Pathologically, these lesions show the following characteristics:[5] they are nonencapsulated congenital fatty tumors, they infiltrate adjacent muscles and soft tissues, there is an absence of lipoblasts and malignant characteristics, fibrous elements are present, and there is hypertrophy of subjacent bone. These lesions can be diagnosed based on the clinical and imaging features.

Plain radiographs show hypertrophy of facial bones and soft tissue swelling. USG may show adipose tissue but cannot delineate the real extent of the lesion.[2] CT scan and MRI are the modalities of choice as they can identify the fat content of the lesions and delineate their extent. CT scan shows fat-density lesions with exquisite detail. The intervening fibrous elements can give a feathery pattern or inhomogeneous character.[4,6] MRI, with its multiplanar capability, is superior to CT. It can depict the exact extent of the lesion. The lesions are inhomogeneously hyperintense on T1W images. Muscle and bony involvement are better seen with MRI. Biopsy may not be required if the typical findings are seen on MRI.[2]

The differential diagnoses[7] include the Proteus syndrome, encephalocutaneous lipomatosis, and vascular malformations (hemangioma). Proteus syndrome is characterized by overgrowth of tissues from all three germ layers. The spectrum consists of hemihypertrophy, facial hamartomas, macrodactyly, and hyperkeratotic rugae of the soles. Encephalocutaneous lipomatosis is characterized by lipomas of the scalp and central nervous system as well as focal alopecia. Hemangiomas and other vascular malformations are compressible lesions that are easily differentiated clinically; they appear isointense on T1W and hyperintense on T2W images and can show flow voids.

The treatment modalities available are liposuction and excision. This is done for cosmetic reasons. Although these tumors are benign, the rate of recurrence is very high after surgical excision.[2,5]

In conclusion, congenital infiltrating lipomatosis of the face is a rare benign condition occurring in childhood. It is characterized by diffuse infiltration of fat in the subcutaneous and muscle planes and bony hypertrophy. Clinical examination and imaging, especially MRI, can establish the diagnosis. Surgery is done for cosmetic purpose.
